# Evidence-Based Strategies for the Prevention of Cardiac Implantable Electronic Device Infections: An Up-to-Date Narrative Review

**DOI:** 10.3390/medicina62050991

**Published:** 2026-05-19

**Authors:** Mantė Agnė Rimkienė, Diana Sudavičienė, Gediminas Račkauskas, Paulius Jurkuvėnas, Veronika Gorevska, Julius Stukas, Germanas Marinskis

**Affiliations:** 1 Clinic of Cardiac and Vascular Diseases, Institute of Clinical Medicine, Faculty of Medicine, Vilnius University, 01513 Vilnius, Lithuania; diana.sudaviciene@santa.lt (D.S.); gediminas.rackauskas@santa.lt (G.R.); paulius.jurkuvenas@santa.lt (P.J.); julius.stukas@mf.stud.vu.lt (J.S.); germanas.marinskis@santa.lt (G.M.); 2 Department of Cardiac Arrhythmias, Vilnius University Hospital Santaros Klinikos, 08406 Vilnius, Lithuania; veronika.gorevska@santa.lt

**Keywords:** cardiac implantable electronic device, CIED infection, infection prevention, perioperative management, antibiotic prophylaxis, device-related infection, electrophysiology, surgical site infection

## Abstract

*Background and Objectives*: Cardiac implantable electronic device (CIED) infections remain among the most serious complications of pacemaker, implantable cardioverter-defibrillator, and cardiac resynchronization therapy procedures. They are associated with substantial morbidity, mortality, prolonged hospitalization, system extraction, long-term antimicrobial therapy, and increased healthcare costs. As most infections arise from perioperative contamination or procedure-related complications, prevention has become a major priority in contemporary electrophysiology practice. This review aimed to summarize current evidence on the prevention of CIED infections, with particular emphasis on modifiable risk factors and perioperative preventive measures. *Materials and Methods*: A focused narrative review was undertaken using targeted searches of PubMed/MEDLINE and Scopus, supplemented by major international guideline and consensus documents, with priority given to contemporary guidelines, randomised trials, meta-analyses, and major observational studies relevant to CIED infection prevention. *Results*: Prevention of CIED infection requires a structured, multifactorial approach spanning the entire procedural pathway. Key preventive strategies include careful reassessment of device indication, individualized device selection, correction of modifiable risk factors, postponement of elective implantation in the presence of active infection, appropriate perioperative antibiotic prophylaxis, and optimized management of anticoagulant and antiplatelet therapy to minimize pocket hematoma. Additional relevant measures include meticulous skin antisepsis, limitation of temporary invasive devices and unnecessary hardware, appropriate venous access selection, careful generator pocket creation and wound closure, and avoidance of early reintervention whenever feasible. Antibacterial envelopes may reduce major CIED infections in selected high-risk patients, whereas routine escalation of preventive measures without proven benefit is not supported. *Conclusions*: CIED infection prevention is inherently multifactorial and depends on the consistent application of evidence-based measures before, during, and after device implantation. Rigorous control of modifiable risk factors, prevention of pocket hematoma, appropriate antimicrobial prophylaxis, and meticulous procedural technique remain the cornerstones of effective infection prevention in patients undergoing CIED procedures.

## 1. Introduction

Cardiac implantable electronic devices (CIEDs) are a cornerstone of contemporary management of bradyarrhythmias, heart failure, and prevention of sudden cardiac death, providing substantial improvements in survival and quality of life [1]. Over recent decades, implantation rates have risen markedly, driven by expanding guideline indications, population aging, and an increasing burden of comorbidities [2,3]. This widespread use of pacemakers, implantable cardioverter-defibrillators, and cardiac resynchronization therapy devices has, however, been accompanied by a growing burden of device-related infections. Although earlier reports described infection rates below 1%, contemporary data demonstrate an increase over time, with incidences ranging from 1% to 7% according to device type and procedural complexity, exceeding the growth in implantation rates [2,4].

CIED infections are associated with substantial morbidity and mortality and typically require complete system extraction, prolonged hospitalization, and long-term antimicrobial therapy, resulting in considerable clinical and economic consequences [5,6]. The economic burden of CIED infection is substantial and varies across healthcare systems. Published cost estimates range from approximately €11,440 to €41,496 per infection episode in European settings [7,8] and from approximately $45,512 to $57,332 in the United States [9,10]. Treatment costs depend largely on infection extent, therapeutic strategy, and length of hospitalization, and are higher when systemic infection, device extraction, prolonged antimicrobial therapy, or reintervention are required [8,10,11,12].

The prognostic impact of CIED infection also depends on both timing and clinical extent. Pooled overall mortality following CIED infection has been reported at 13.7% [13]. A prospective observational cohort study recently demonstrated that early systemic infections (≤3 months) and delayed localized infections (3–12 months) were associated with an approximately three-fold increase in mortality, whereas delayed systemic infections carried the highest risk of death, with a 9.3-fold increase in mortality [14]. These findings emphasize that systemic involvement and delayed presentation identify particularly high-risk infection phenotypes and further support early recognition, appropriate management, and effective preventive strategies.

In this context, prevention of CIED infection has become a central priority in modern electrophysiology practice. This review summarizes current evidence-based strategies for the prevention of CIED infections, with a particular focus on perioperative and procedure-related measures.

## 2. Methods of the Review

This article was designed as a structured narrative review, prepared with attention to SANRA principles, to summarise clinically relevant strategies for the prevention of cardiac implantable electronic device (CIED) infection. Relevant English-language publications were identified through targeted searches of PubMed/MEDLINE and Scopus, supplemented by major international guideline and consensus documents, with a primary focus on literature published from 2015 to 2026; earlier landmark studies were retained selectively where they remained foundational. Representative search terms included “cardiac implantable electronic device”, “CIED infection”, “pacemaker infection”, “implantable cardioverter-defibrillator infection”, “infection prevention”, “antibiotic prophylaxis”, “pocket haematoma”, “anticoagulation”, “skin antisepsis”, “antibacterial envelope”, “temporary pacing”, “venous access” and “surgical site infection”. Publications were selected for direct relevance to CIED infection prevention, with priority given to contemporary guidelines and consensus statements, randomised trials, meta-analyses, and large observational studies; case reports, small case series, editorials, and non-CIED-specific literature were not prioritised unless clinically essential. Evidence was synthesised narratively within predefined thematic domains, with greater interpretive weight assigned to higher-level and clinically applicable evidence. Because this was a structured narrative review rather than a systematic or scoping review, no PRISMA flow diagram, duplicate screening process, formal risk-of-bias assessment, or quantitative synthesis was undertaken. The main preventive interventions discussed in this review and the overall strength of supporting evidence are summarized in Table 1.

## 3. Pathogenesis and Risk Factors of CIED Infection

Cardiac implantable electronic device infections most commonly result from perioperative contamination of the device pocket, leads, or generator at the time of implantation, whereas haematogenous seeding from distant infections represents a less frequent mechanism [15].

Staphylococci represent the dominant pathogens in CIED infections, reflecting their skin commensal origin and ability to adhere to device surfaces [1,4,6,7]. Coagulase-negative staphylococci are frequently isolated, particularly in more indolent pocket infections, whereas *Staphylococcus aureus* is especially relevant in acute presentations, bloodstream infection, and systemic CIED infection [1,4,6,16]. Both coagulase-negative staphylococci and *S. aureus* may form biofilms on device components, limiting antibiotic penetration, promoting bacterial persistence, and contributing to the difficulty of eradicating established infection without complete system removal [15,17].

This microbiological profile supports prevention strategies that primarily target skin flora and early perioperative contamination, including appropriate antibiotic prophylaxis, meticulous antisepsis, and avoidance of pocket haematoma or reintervention [1,6,15].

Major patient-, device-, and procedure-related risk factors for CIED infection are summarized in Table 2.

## 4. Evidence-Based Preventive Strategies

CIED infection prevention relies on a structured, multi-step approach targeting modifiable risk factors across the entire procedural pathway. In this section, we review two major groups of evidence-based interventions: (1) pre-procedural patient optimization and (2) peri-operative procedural strategies. Together, these measures aim to minimize bacterial contamination, reduce pocket hematoma formation, and improve overall clinical outcomes.

### 4.1. Pre-Procedural Patient Optimization

#### 4.1.1. Reassessment of Device Indication

Careful consideration should be given to whether the anticipated benefit of device implantation outweighs the procedural and long-term infectious risks in each individual patient. Although guideline-directed indications remain the foundation of decision-making, real-world data suggest that a substantial proportion of patients undergoing device extraction for infection do not ultimately require reimplantation. Observational studies report that approximately 14–24% of patients may remain device-free after system removal, reflecting initial implantation in the context of potentially reversible or secondary causes [23,24,25]. These findings underscore the importance of thorough preprocedural reassessment, including exclusion of reversible etiologies (e.g., drug-induced bradycardia, metabolic disturbances, acute ischemia) and individualized risk–benefit evaluation. Importantly, excess mortality observed in this population appears to be primarily driven by comorbid conditions and infectious complications rather than untreated bradyarrhythmia or device withdrawal itself, suggesting that, in carefully selected patients, a strategy of non-systematic reimplantation with close clinical surveillance may be safe [24,25,26].

#### 4.1.2. Individualized Device Selection

Careful consideration of device type is warranted in the presence of non-modifiable patient-related risk factors, as increasing system complexity, particularly implantation of ≥2 transvenous leads or dual-chamber devices, has been consistently associated with a significantly higher risk of device-related infection (Table 2). However, device simplification should not be interpreted as an independently proven infection-prevention intervention, but rather as a risk-mitigation principle to be considered when clinically appropriate.

Evidence from randomized pacing trials provides indirect support for the clinical acceptability of simpler pacing strategies in selected populations, although these studies were not designed to evaluate infectious outcomes. Extended register-based follow-up of the DANPACE trial, including all 1384 patients with sick sinus syndrome randomized to AAIR or DDDR pacing, demonstrated comparable long-term mortality over a mean follow-up of 8.9 years (adjusted HR 1.03; 95% CI 0.90–1.19; *p* = 0.65), with no differences in hospitalization for atrial fibrillation, stroke, or heart failure [27]. Similarly, in the UKPACE trial, which enrolled 2021 patients aged ≥70 years undergoing first pacemaker implantation for high-grade atrioventricular block, single-chamber ventricular pacing was not associated with inferior all-cause mortality compared with dual-chamber pacing over a median follow-up of approximately 4.6 years (HR 0.96; 95% CI 0.83–1.11), nor were differences observed in major cardiovascular outcomes [28].

Accordingly, these randomized data should be interpreted as supporting the feasibility of avoiding unnecessary system complexity in carefully selected patients, rather than proving a direct infection-prevention benefit. In this context, current European Society of Cardiology (ESC) consensus on conduction system pacing (CSP) acknowledges CSP as a reasonable alternative to conventional biventricular pacing in selected patient populations. CSP may avoid coronary sinus lead placement in selected scenarios or provide an alternative when conventional left ventricular lead implantation is not feasible; however, it does not necessarily reduce the total amount of implanted hardware, particularly in CRT-D or ICD systems. In selected patients at elevated infectious risk, such alternatives may still be clinically relevant when they allow an appropriate therapeutic effect without unnecessary system complexity. Thus, any potential infection-prevention advantage of CSP should be considered indirect and context-dependent rather than an established benefit [29].

In selected patients, non-transvenous device strategies may further reduce intravascular hardware burden. Leadless pacemakers avoid a subcutaneous generator pocket and transvenous leads and may be considered when pacing requirements can be adequately met by a leadless system. Earlier evidence mainly involved single-chamber ventricular leadless pacing, including observational data from the Micra post-approval registry showing favourable infection-related outcomes in patients with recent or pre-existing CIED infection undergoing leadless pacemaker implantation [30]. More recently, dual-chamber leadless pacing has expanded potential indications beyond isolated ventricular pacing, with the AVEIR DR i2i study demonstrating short-term safety and reliable atrioventricular synchrony in patients with conventional dual-chamber pacing indications [31]. However, leadless pacing should still be considered an indication-dependent alternative rather than a universal infection-prevention strategy, as long-term evidence, retrieval/replacement considerations, availability, and patient-specific pacing needs remain important limitations.

Taken together, these data support an individualized indication-driven approach to device selection. In patients at high infectious risk, less complex or non-transvenous systems may be considered when they adequately meet pacing or defibrillation requirements; however, such decisions should remain subordinate to the primary electrophysiological indication, expected clinical benefit, and long-term therapeutic needs.

#### 4.1.3. *Staphylococcus aureus* Screening and Decolonization

Nasal carriage of *Staphylococcus aureus* has been associated with an increased risk of surgical site infection (SSI) in multiple surgical populations, providing a rationale for targeted preprocedural screening and decolonization strategies [32].

Targeted decolonization using intranasal mupirocin combined with chlorhexidine body washes, initiated several days prior to implantation, may reduce bacterial burden and potentially lower infection risk [32]. However, universal screening is not routinely recommended, and implementation should be tailored to institutional prevalence patterns and patient-specific risk factors.

Screening for *S. aureus* colonization may be considered in selected high-risk patients or in institutions with a high prevalence of methicillin-resistant strains, in line with current consensus recommendations [1,33].

#### 4.1.4. Preoperative Bathing

With regard to preoperative patient preparation, the World Health Organization guideline development group considers preoperative bathing or showering to be good clinical practice aimed at reducing overall skin bacterial load prior to surgery. However, moderate-quality evidence indicates that bathing with chlorhexidine-containing soap offers no additional benefit over plain soap in reducing SSI rates [33]. In selected cases with known colonization or active dermatologic conditions (e.g., chronically colonized diabetic foot ulcers), targeted decolonization guided by microbiological identification and susceptibility testing may be considered [34].

#### 4.1.5. Hair Removal

Hair removal has been evaluated as part of preoperative skin preparation, with a Cochrane review demonstrating no significant reduction in SSI rates compared with no hair removal [35]. Accordingly, routine hair removal is not recommended. When hair removal is necessary to facilitate surgical exposure or wound management, current guidelines advise the use of clippers rather than razors, performed outside the operating room and as close to the time of surgery as possible [33]. This approach minimizes microscopic skin trauma and preserves skin barrier integrity, which is critical for reducing the risk of perioperative contamination.

#### 4.1.6. Glycemic Control

Perioperative glycemic control represents an important modifiable factor in the prevention of surgical site infections. Based on a systematic evaluation of available observational studies and randomized controlled trials, the World Health Organization recommends the use of protocols for perioperative blood glucose control in adult patients undergoing surgical procedures, irrespective of diabetic status. Evidence indicates that intensive perioperative blood glucose control is associated with a significant reduction in SSI rates, with target glucose levels of 6.1 mmol/L (≤110 mg/dL) and an upper acceptable range of 6.1–8.3 mmol/L (110–150 mg/dL) [33].

Although diabetes mellitus is a recognized risk factor for CIED infection, specific glycated haemoglobin (HbA1c) thresholds associated with increased infection risk have not been clearly defined in the device literature. Evidence from broader surgical populations suggests that infection risk begins to increase when HbA1c exceeds values above approximately 7% [36].

Accordingly, optimization of perioperative and long-term glycemic control prior to elective CIED implantation should be considered whenever clinically feasible.

#### 4.1.7. Immunosuppressive Agents

Current expert consensus does not support routine discontinuation of immunosuppressive therapy prior to device implantation for the purpose of preventing surgical site infection, as no relevant evidence demonstrates benefit [33]. Abrupt interruption of immunosuppressive agents may instead increase the risk of disease flare or organ rejection. However, when clinically feasible, elective procedures should be deferred until the patient is maintained on the lowest effective dose of immunosuppression, particularly in the case of chronic corticosteroid therapy [34]. Optimization of immunosuppressive regimens and individualized timing of implantation should be undertaken in close collaboration with the treating specialist to balance infection risk against the risk of underlying disease destabilization.

#### 4.1.8. Anticoagulation and Antiplatelets

Periprocedural management of oral anticoagulation and antiplatelet therapy is a critical determinant of pocket haematoma formation, one of the strongest procedure-related predictors of CIED infection, associated with more than an eight-fold increase in infection risk in pooled analyses (OR 8.46; 95% CI 4.01–17.86) [16].

In patients treated with vitamin K antagonists, the BRUISE CONTROL-1 trial demonstrated that uninterrupted warfarin therapy during CIED implantation significantly reduced the risk of clinically significant pocket haematoma compared with heparin bridging (3.5% vs. 16.0%; RR 0.19; 95% CI 0.10–0.36; *p* < 0.001) [37]. Continuation of warfarin within the therapeutic INR range is therefore recommended (Table 3).

In contrast, evidence regarding direct oral anticoagulants (DOACs) is less definitive. The BRUISE CONTROL-2 trial demonstrated no difference in clinically significant pocket haematoma rates between uninterrupted DOAC therapy and temporary interruption (2.1% vs. 2.1%; *p* = 0.97) [38]. Current guideline documents therefore indicate that either continuation or temporary interruption may be reasonable, depending on renal function, bleeding risk, thromboembolic risk, and procedural complexity [39,40]. When DOAC interruption is selected, timing should be individualized based on renal function, bleeding risk, and procedural complexity with the procedure ideally performed at trough DOAC levels (Table 3) [40].

Regarding antiplatelet therapy, aspirin monotherapy can usually be continued during CIED implantation, whereas dual antiplatelet therapy and combined oral anticoagulant–antiplatelet therapy are associated with a substantially increased risk of bleeding and pocket haematoma. Observational studies have demonstrated that dual antiplatelet therapy, most commonly aspirin combined with a P2Y12 inhibitor such as clopidogrel, is associated with approximately four- to five-fold higher risk of pocket haematoma compared with patients not receiving antiplatelet therapy [41,42]. According to ESC guidelines, P2Y12 inhibitors should, whenever clinically feasible, be discontinued prior to CIED implantation, particularly in patients receiving concomitant oral anticoagulation (Table 3) [39].

#### 4.1.9. Antibiotic Prophylaxis

Antibiotic prophylaxis remains the cornerstone of infection prevention in cardiac implantable electronic device implantation, as most CIED infections originate from perioperative contamination with skin flora at the time of implantation. Perioperative antibiotic administration has been shown to significantly reduce the incidence of CIED infection compared with no antibiotic therapy, with randomized trials and pooled analyses demonstrating relative risk reductions of up to approximately 80% [17,19,43].

Perioperative antibiotic prophylaxis should primarily target skin flora, which represent the predominant source of CIED infections [4]. Current surgical prophylaxis guidelines recommend first- or second-generation cephalosporins as first-line agents for cardiac device implantation, with alternative agents reserved for patients with documented β-lactam allergy or specific risk factors such as MRSA colonization [18]. Detailed recommendations regarding agent selection, dosing, timing, and redosing intervals are summarized in Table 4.

Incremental antibiotic strategies have been investigated as adjunctive measures to reduce bacterial burden and lower the risk of CIED infection. In the PADIT trial (Prevention of Arrhythmia Device Infection Trial), which included 19,603 patients, an incremental prophylactic strategy consisting of preprocedural cefazolin plus vancomycin, intraprocedural bacitracin pocket irrigation, and a 2-day course of postoperative oral cephalexin did not significantly reduce CIED infection rates compared with conventional single-dose preprocedural cefazolin prophylaxis (0.78% vs. 1.03%; OR 0.77; 95% CI 0.56–1.05; *p* = 0.10) [44]. Based on these findings, current international guidelines do not recommend routine use of incremental or extended perioperative antibiotic strategies, given the lack of proven clinical benefit. Moreover, unnecessary exposure to broad-spectrum or prolonged antibiotic regimens is a well-recognized contributor to antimicrobial resistance, underscoring the importance of antibiotic stewardship in CIED implantation.

#### 4.1.10. Limiting Temporary Invasive Devices

Temporary invasive devices represent an important and potentially modifiable source of bacteremia in patients undergoing CIED implantation. Extensive healthcare-associated infection literature demonstrates that indwelling vascular and urinary catheters increase bloodstream infection risk in proportion to catheter dwell time.

Central venous catheters are strongly associated with bloodstream infections, with reported incidence densities typically ranging from approximately 1–2 per 1000 catheter-days in contemporary hospital settings [45]. Peripheral intravenous catheters are associated with substantially lower rates of bloodstream infection (0.044 per 1000 catheter-days), whereas local infectious complications occur more frequently, at approximately 0.65 per 1000 catheter-days [46]. Particular attention should be paid to peripheral catheters placed in the ipsilateral upper limb, as venous access for CIED leads is commonly obtained via the cephalic, axillary, or subclavian vein, which are in direct anatomical continuity with peripheral veins of the same arm. Local inflammation, endothelial disruption, or catheter-related bacteremia in the ipsilateral limb may facilitate microbial access to newly implanted hardware.

Urinary catheters likewise represent a frequent source of healthcare-associated infection, with reported incidences of catheter-associated urinary tract infection of approximately 3–7 per 1000 catheter-days depending on the clinical setting [47]. The daily risk of bacteriuria increases with dwell time and may lead to secondary bacteremia. In the presence of recently implanted intracardiac hardware, even transient bacteremia may promote device colonization.

Accordingly, systematic reassessment of all temporary invasive devices prior to CIED implantation, avoidance of ipsilateral peripheral venous access when feasible, and prompt removal of unnecessary vascular or urinary catheters constitute pragmatic measures to reduce peri-procedural infectious risk.

#### 4.1.11. Timing of Procedure

Optimal timing of CIED implantation represents an important modifiable factor in infection prevention. Implantation in the presence of active infection substantially increases the risk of device-related infection (Table 2), particularly when additional risk factors for infection are present. Fever within 24 h prior to implantation has been associated with a five- to six-fold increase in infection risk [48], underscoring the importance of deferring elective procedures during active infection. Current guidelines therefore recommend postponing elective CIED implantation until the patient has been afebrile for at least 24 h and clinical signs of infection have resolved [1,39]. Improvement or normalization of inflammatory markers, including C-reactive protein and white blood cell count, may provide additional reassurance of procedural safety, although no specific laboratory thresholds have been formally established.

Moreover, CIED implantation is contraindicated in the presence of active infective endocarditis (IE) unless life-threatening bradyarrhythmia cannot be managed by alternative means [1]. With respect to reimplantation following IE, expert consensus recommends that reimplantation should be deferred until blood cultures remain negative for at least 72 h in the absence of vegetations, and for at least 2 weeks when valvular vegetations are present [49]. When feasible, reimplantation should be performed on the contralateral side [1].

#### 4.1.12. Temporary Pacing

Temporary pacing has also been identified as an important procedure-related risk factor for CIED infection. In pooled analyses including both prospective and retrospective studies, the use of temporary pacing was associated with a more than two-fold increase in infection risk (OR 2.31; 95% CI 1.36–3.92) [16]. However, available evidence does not allow stratification of this risk according to venous access site. Femoral access is generally considered less favourable and should be avoided whenever feasible. Whenever possible, pharmacological chronotropic support or temporary pacing via the intended permanent device access should be preferred, and the duration of temporary pacing should be minimised by early transition to permanent device implantation.

### 4.2. Intra-Procedural Preventive Strategies

#### 4.2.1. Skin Preparation and Antisepsis

Given that most CIED infections originate from perioperative contamination with skin flora, effective preoperative skin preparation represents a key component of infection prevention strategies. Effective antiseptic skin preparation becomes a central component of perioperative infection prevention. Neither ESC nor American Heart Association (AHA) guidelines provide agent-specific recommendations for preoperative skin antisepsis, instead emphasizing meticulous aseptic technique. In routine clinical practice, a limited number of antiseptic agents are commonly used for preoperative skin preparation, with alcohol-based formulations predominating (Table 5). Randomized clinical trials in general surgical populations have demonstrated superior efficacy of alcohol-based antiseptic solutions compared with aqueous preparations [50,51]. However, when alcohol-based regimens are compared, differences between specific antiseptic agents appear limited. In the CLEAN 2 trial, a large multicenter randomized study including 3242 cardiac surgery patients, no significant difference in SSI rates was observed between chlorhexidine–alcohol and alcohol-based povidone–iodine (4.0% vs. 3.3%; risk difference 0.74; 95% CI −0.55 to 2.03; *p* = 0.26) [52]. Similarly, in a multicenter randomized trial including 2272 patients undergoing cardiac resynchronization therapy procedures, device-related infection rates did not differ significantly between alcohol-based chlorhexidine and alcohol-based povidone–iodine (2.9% vs. 3.9%; adjusted subhazard ratio 0.75; 95% CI 0.48–1.20; *p* = 0.23) [53]. Taken together, these data suggest that, provided an alcohol-based formulation is used, the choice between specific antiseptic agents is unlikely to have a major impact on infection risk.

#### 4.2.2. Adhesive Incise Drapes

According to a Cochrane systematic review, iodine-impregnated adhesive incise drapes did not reduce surgical site infection rates compared with no drape (RR 1.03, 95% CI 0.66–1.60), whereas non-antimicrobial adhesive drapes were associated with a significantly higher incidence of SSI (RR 1.23, 95% CI 1.02–1.48) [54]. These findings are further supported by Eckler et al., who demonstrated an increased risk of wound infection associated with non-iodine-impregnated adhesive drapes (RR 1.29, 95% CI 1.02–1.65) [55]. Additional supportive evidence for iodine-impregnated incise drapes was provided by Bejko et al. in a large cardiac surgery cohort of 5100 patients, where postoperative complications were significantly more frequent in the standard drape group compared with the iodine-impregnated drape group (6.5% vs. 1.9%, *p* = 0.001). Notably, inadequate drape-to-skin adhesion was identified as a potential contributing mechanism, with complete adhesion maintained in fewer than 60% of patients in the standard drape group [56].

Importantly, cardiac implantable electronic device (CIED)-specific randomized data have recently emerged in selected high-risk settings. In a randomized clinical trial including 418 patients undergoing repeat CIED implantation, Aydin et al. demonstrated that iodine-impregnated adhesive incise drapes were associated with reduced pocket bacterial contamination and fewer adjudicated CIED infections at 1-year follow-up, suggesting a potential protective effect in this high-risk population [57].

From a guideline perspective, the World Health Organization explicitly recommends against the routine use of adhesive incise drapes for the prevention of SSI. European Society of Cardiology guidelines acknowledge their frequent use in clinical practice, but emphasize the lack of evidence supporting their effectiveness and caution that non-iodophor drapes may increase infection risk [1], whereas American Heart Association guidelines do not provide specific recommendations regarding the use of adhesive incise drapes in CIED implantation [1,33]. Overall, available data and current guidelines do not justify the routine use of adhesive incise drapes for SSI prevention in CIED implantation. However, available evidence suggests that iodine-impregnated drapes may be superior to no drape in selected high-risk settings, whereas non-antimicrobial drapes should be avoided.

#### 4.2.3. Selection of Venous Access Route

The choice of venous access during CIED implantation influences acute procedural safety and long-term lead durability, thereby affecting cumulative infection risk, as repeat intervention remains a major determinant of device-related infection.

Evidence consistently suggests that traditional subclavian puncture (SP) is associated with less favorable mechanical outcomes compared with extrathoracic approaches. In a comprehensive meta-analysis by Benz et al., including more than 30,000 patients, cephalic vein cutdown (CVC) was associated with significantly lower rates of pneumothorax and overall lead failure compared with SP [58]. In an observational study by Chan et al. involving 409 patients, the use of SP, rather than axillary puncture (AP), was identified as the only independent predictor of pacemaker lead failure, indicating inferior long-term lead performance with the subclavian approach [59]. In a retrospective cohort of 1673 ICD implantations, Morani et al. reported a low overall lead failure rate with axillary venous access [60]. Importantly, the presence of three transvenous leads within the same vein independently predicted lead failure, underscoring the importance of minimizing intravascular hardware burden in addition to selecting an appropriate access strategy.

Although CVC demonstrated lower primary venous cannulation success rates in the study by Chan et al. [59], available evidence indicates that both axillary and cephalic approaches are associated with more favorable long-term lead durability compared with SP. In the absence of direct infection-specific data, avoidance of intrathoracic subclavian access and minimization of intravascular lead burden appear mechanistically justified strategies to reduce downstream reintervention and cumulative infection risk.

#### 4.2.4. Venous Puncture Technique

Although current evidence does not demonstrate a clear difference in CIED infection outcomes between ultrasound- and fluoroscopy-guided techniques, strategies that minimize vascular trauma and access-site complications may be clinically relevant within a comprehensive infection prevention framework.

Randomized evidence indicates that ultrasound-guided and fluoroscopy-guided axillary venous access provide comparable procedural success and short-term safety during CIED implantation. In the ZEROFLUOROAXI randomized trial (*n* = 384), no significant differences were observed between techniques in rates of pneumothorax, hemothorax, pocket hematoma, pocket infection, lead dislodgement, or 30-day mortality [61]. First-attempt success and total procedural duration were likewise similar. The principal difference was a significantly lower incidence of inadvertent axillary arterial puncture with ultrasound guidance (6% vs. 17%; *p* = 0.004), accompanied by reduced fluoroscopy exposure. Subgroup analyses stratified by body mass index (<30 vs. ≥30 kg/m^2^) demonstrated consistent performance across BMI categories.

These findings are supported by prospective observational data from a cohort of 1000 patients reported by Perna et al., in which procedural success and overall safety were comparable across cephalic, subclavian, and ultrasound-guided axillary approaches [62]. Ultrasound-guided access, however, was associated with fewer puncture attempts and shorter time to venous access compared with subclavian puncture, while also reducing access time relative to cephalic cutdown, without prolonging total implantation time.

While major complication rates appear comparable between techniques, ultrasound guidance may offer practical advantages by reducing vascular trauma and inadvertent arterial puncture, factors that are mechanistically relevant in minimizing access-site complications within an infection prevention strategy.

#### 4.2.5. Absorbable Antibiotic-Eluting Envelope

In contrast, the use of an absorbable antibiotic-eluting envelope has demonstrated a clinically meaningful reduction in CIED infection risk in selected patient populations. In the WRAP-IT trial, a large randomized controlled study including 6983 patients undergoing CIED pocket revision, generator replacement, system upgrade, or de novo implantation of a cardiac resynchronization therapy defibrillator, the use of a minocycline and rifampicin eluting antibacterial envelope was associated with a 40% relative reduction in major CIED infections compared with standard infection prevention strategies alone (HR 0.60; 95% CI 0.36–0.98; *p* = 0.04) [63]. Importantly, use of the antibacterial envelope was not associated with an increase in procedure-related complications or all-cause mortality. As these results are derived predominantly from higher-risk procedures, contemporary guideline documents recommend consideration of antibacterial envelopes in patients at increased risk for CIED infection, whereas routine use in low-risk primary implantations is not supported [1,6].

Economic analyses based on WRAP-IT data suggest that the antibacterial envelope is cost-effective in selected high-risk populations, particularly in patients undergoing device replacement, upgrade, or revision, where baseline infection risk is higher. However, cost-effectiveness appears less favorable in low-risk de novo implantations, reinforcing a risk-stratified approach to its use [64].

#### 4.2.6. Generator Pocket Size and Depth

Generator pocket creation may influence postimplantation complications that predispose to infection. Excessive tissue tension in an undersized pocket may impair local perfusion and wound healing, whereas oversized pockets can create dead space, predisposing to hematoma or seroma formation, well established predictors of subsequent CIED infection (Table 2). Prevention of pocket hematoma has therefore been emphasized in expert consensus statements as a key component of infection risk reduction [1,6]. Although direct comparative studies defining optimal pocket dimensions are lacking, careful tailoring of pocket size to generator dimensions, meticulous hemostasis, and minimization of unnecessary tissue dissection are justified to reduce hematoma and other wound-related complications.

Beyond pocket dimensions, the depth of generator implantation may influence local complication rates, particularly in patients with limited soft tissue coverage. Low body mass index has been associated with an increased risk of pocket-related complications, including hematoma and device erosion, both recognized risk factors of CIED infection [65]. While most CIEDs are implanted in a prepectoral subcutaneous pocket, submuscular placement has been used as an alternative in such high-risk cases. In a prospective cohort of underweight patients, submuscular generator implantation was associated with fewer early infectious complications compared with subcutaneous placement; notably, no infections were observed in the submuscular group, whereas superficial wound infections occurred exclusively in the subcutaneous cohort [66]. In this context, enhanced tissue coverage achieved through submuscular implantation may reduce mechanical stress on the overlying skin and mitigate erosion risk. However, the study was non-randomized, limited in sample size, and restricted to short-term follow-up. A prospective randomized trial (POCKET) comparing subcutaneous versus intramuscular pocket placement has been initiated, underscoring ongoing interest in the clinical implications of pocket depth, although results regarding infection outcomes remain forthcoming [67].

Although robust comparative data on long-term infection reduction remain limited, deeper pocket implantation may be considered in carefully selected patients with low BMI or fragile soft tissue, balancing potential benefits against increased procedural complexity.

#### 4.2.7. Pocket Irrigation Strategies

Contemporary ESC and AHA guidelines recommend vigorous pocket irrigation, reflecting its role in mechanically reducing contaminants, while routine local instillation of antibiotics or antiseptics is not recommended [1,6]. Although a variety of antiseptic solutions have been used worldwide during CIED implantation, only taurolidine-containing antimicrobial adjuncts are currently licensed for intrapocket use in this setting, with available safety data supporting their intended application. However, the existing evidence base remains limited and is derived primarily from observational studies, and therefore does not support routine recommendation at a guideline level. In contrast, the use of locally instilled antibiotics or antiseptics, including ethanol-based solutions, chlorhexidine (aqueous or alcoholic), hydrogen peroxide, hypochlorous acid, octenidine, povidone–iodine, and polyhexanide, remains off-label and is limited by concerns regarding cytotoxicity, impaired wound healing, allergic reactions, or potential damage to device components [34]. Given the lack of consistent evidence demonstrating infection reduction and the potential for local tissue or hardware-related adverse effects, routine use of non-licensed antiseptic irrigants during CIED procedures cannot be recommended.

#### 4.2.8. Suture Materials and Wound Closure Techniques

Suture selection and closure strategy may contribute to wound healing and indirectly influence infection risk following CIED implantation. Material characteristics affect bacterial adherence and local tissue response, which are particularly relevant in procedures involving permanent intravascular hardware. In general, synthetic absorbable monofilament sutures are preferred for layered wound closure due to lower bacterial colonization compared with braided materials, whereas device fixation requires non-absorbable sutures that provide reliable tensile strength and durable mechanical support [68,69]. Absorbable braided sutures should be avoided in contaminated or potentially contaminated wounds because of their higher propensity for bacterial retention [70]. Further structural and biological properties relevant to infection risk are summarized in Table 6.

Antimicrobial-coated sutures, most commonly triclosan-impregnated, have been evaluated in multiple randomized clinical trials and meta-analyses. A large updated meta-analysis of 31 RCTs including nearly 18,000 patients found that use of triclosan-containing sutures was associated with a significantly lower risk of SSI compared with non-coated sutures (RR 0.75; 95% CI 0.65–0.86), with moderate certainty of evidence [71]. Smaller meta-analyses of 25 RCTs have similarly demonstrated a reduction in SSI risk with triclosan-coated sutures (RR ~0.73; 95% CI 0.65–0.82) [72].

Closure technique may also be relevant. Interrupted sutures allow individualized tension adjustment and precise wound edge approximation, while continuous sutures enable rapid and uniform closure but depend on the integrity of a single running strand [73]. Subcuticular techniques may reduce superficial irritation by minimizing transcutaneous foreign material and improve cosmetic outcomes [74].

Overall, no single suture material or closure technique has been conclusively shown to eliminate infection risk in CIED implantation. Although antimicrobial-coated sutures have demonstrated a reduction in surgical site infection in broader surgical populations, CIED-specific evidence remains limited. Therefore, emphasis should remain on atraumatic tissue handling, meticulous hemostasis, and tension-free layered closure, while suture selection should be integrated into a comprehensive perioperative infection prevention strategy.

#### 4.2.9. Timing of Reintervention

Early reoperation has been consistently identified as one of the strongest procedure-related risk factors for CIED infection. In a large registry analysis including over 200,000 ICD implantations, adverse events during implantation requiring reintervention were associated with a significantly increased risk of device infection (OR 2.69; 95% CI 2.30–3.15) [75]. This association is supported by a systematic review and meta-analysis, in which early lead repositioning was associated with a substantially increased risk of CIED infection (pooled OR 6.37; 95% CI 3.29–12.31) [16]. However, it remains unclear whether delaying reintervention, when clinically feasible, is associated with a lower risk of subsequent CIED infection.

#### 4.2.10. Same-Day CIED Implantation and Discharge

Same-day discharge after cardiac implantable electronic device implantation has emerged as a feasible strategy to improve resource utilization without compromising patient safety. In the E-MOTION randomized trial, early mobilization 3 h after CIED implantation was not associated with higher rates of lead dislodgement or early postprocedural complications compared with standard 24-h immobilization, supporting the safety of shorter postimplant monitoring in selected patients [76]. Large observational data further support the safety of expedited discharge protocols: in a cohort of 4543 procedures, same-day discharge was not associated with increased 90-day infection or complication rates compared with overnight hospitalization [77]. Prospective registry data also demonstrate comparable complication and infection rates between patients discharged the same day and those observed overnight, along with reduced hospital stay and high patient satisfaction [78].

Taken together, available evidence suggests that same-day implantation and discharge may be safely implemented in carefully selected patients with uncomplicated procedures and structured follow-up.

#### 4.2.11. Prolonged Procedure Duration

Prolonged procedural duration is a reported risk factor for CIED infection. Longer operative time may reflect greater procedural complexity, repeated manipulation, tissue trauma, and bleeding, thereby facilitating bacterial contamination of newly implanted hardware.

Meta-analytic evidence supports this association. In a systematic review by Polyzos et al., procedures complicated by CIED infection were on average 9.89 min longer than uncomplicated procedures (95% CI 0.52–19.25) [16]. Registry data further support a duration–risk relationship. Data from the Danish device registry showed that, compared with procedures lasting <30 min, the relative risk (RR) [95% CI] of infection increased progressively with longer procedural duration: 1.54 [1.24–1.91] for procedures lasting 60–90 min, 1.85 [1.36–2.49] for 90–120 min, and 2.42 [1.77–3.33] for procedures lasting >120 min [79].

Data from contemporary randomized trial cohorts further reinforce these findings. In secondary analyses of the WRAP-IT population, prolonged procedure duration was identified as an independent predictor of major CIED infection, with risk models incorporating a threshold of >60 min, particularly in replacement, upgrade, or revision procedures [22]. More recently, the BLISTER score demonstrated that skin-to-skin time ≥ 120 min was independently associated with more than a two-fold increase in infection risk (adjusted HR 2.6; 95% CI 1.6–4.1) [20].

The cumulative evidence indicates an increase in infection risk with longer procedures, particularly beyond two hours. Procedural efficiency and careful planning should therefore be considered integral components of infection prevention.

## 5. Practical Risk-Stratified Perioperative Prevention Pathway

Beyond summarizing individual preventive measures, this review emphasizes the need to integrate patient-related, device-related, and procedure-related risk factors into a practical perioperative prevention pathway. In patients scheduled for CIED implantation, infection prevention should begin with reassessment of device indication and individualized device selection, followed by structured evaluation of modifiable risk factors, optimization of antithrombotic therapy, appropriate antibiotic prophylaxis, and meticulous procedural technique.

Risk stratification tools, including the PADIT and BLISTER scores, may help identify patients at increased risk of CIED infection and guide the selective use of adjunctive preventive strategies, such as antibacterial envelopes [20,63,80]. In selected high-risk patients, alternative device strategies that reduce or avoid transvenous hardware may be considered when they adequately meet the patient’s pacing or defibrillation requirements. However, these strategies should be individualized according to pacing or defibrillation indication, patient anatomy, comorbidities, and long-term treatment needs.

From a practical standpoint, perioperative prevention may be structured into three sequential steps: pre-procedural risk assessment and device planning, intra-procedural contamination and hematoma prevention, and post-procedural monitoring with avoidance of unnecessary early reintervention. Accordingly, CIED infection prevention should be viewed not as a single intervention, but as a risk-stratified bundle of measures applied across the entire procedural pathway.

## 6. Practical Take-Home Messages

○ CIED infection prevention should begin before implantation. Reassess device indication, infection risk, and hardware burden to avoid unnecessary system complexity when clinically appropriate.
○ Do not implant during active infection. Elective CIED procedures should be postponed in patients with fever, active systemic infection, or local infection at the intended implantation site.
○ Perioperative antibiotic prophylaxis should be administered routinely. An appropriately timed pre-incision dose targeting skin flora is recommended for most patients; routine prolonged or intensified antibiotic regimens are not supported.
○ Prevent pocket haematoma. Avoid heparin bridging, continue therapeutic VKA when appropriate, and individualize DOAC and antiplatelet management according to bleeding and thromboembolic risk.
○ Use meticulous and efficient procedural technique. Alcohol-based skin antisepsis, careful venous access selection, precise haemostasis, appropriate pocket creation, tension-free layered wound closure, and avoidance of unnecessary procedural prolongation remain core preventive measures.
○ Minimize temporary pacing, temporary invasive devices, and early reintervention. Each additional invasive step may increase infectious risk and should be avoided whenever feasible.
○ Use adjunctive preventive measures selectively. Antibacterial envelopes may be considered in high-risk CIED procedures, iodine-impregnated adhesive drapes in selected repeat procedures, and antimicrobial-coated sutures as part of optimized wound closure, whereas routine escalation of unproven measures should be avoided.
○ Infection prevention requires a bundled approach: consistent use of multiple evidence-based measures across all stages-before, during, and after the procedure-provides the greatest protection.

## 7. Conclusions

Cardiac implantable electronic device infections are largely preventable and are predominantly driven by perioperative factors. Contemporary evidence demonstrates that consistent application of evidence-based perioperative practices substantially reduces infection risk, while unnecessary escalation of preventive measures without proven benefit should be avoided.

## Figures and Tables

**Table 1:** Summary of preventive interventions, supporting evidence, and practical implications for CIED infection prevention.

Preventive Intervention	Main Supporting Evidence	Strength of Evidence	Practical Implication
Individualized device selection/avoidance of unnecessary hardware complexity	Randomized trials, observational infection-risk data, and consensus documents	Moderate, indirect	Consider less complex systems in selected patients when clinically appropriate and when infectious risk is high
Perioperative glycemic control	Guidelines based on surgical RCTs and observational studies	Moderate, indirect	Optimize perioperative glucose; consider HbA1c optimization if >~7%, recognizing no CIED-specific threshold
Avoidance of pocket hematoma (antithrombotic management)	Randomized controlled trials, observational studies, and guideline documents	High	Continue therapeutic VKA when appropriate; avoid heparin bridging; individualize DOAC and antiplatelet management
Perioperative antibiotic prophylaxis	Randomized controlled trials, meta-analyses, and guideline documents	High	Recommended routinely
Temporary pacing minimization	Observational studies and meta-analyses	Moderate	Minimize use and duration whenever feasible
Limiting temporary invasive devices	Observational studies and infection control data	Low to moderate, indirect	Remove unnecessary catheters; avoid ipsilateral access
Deferral in active infection/fever	Guideline/consensus documents and observational infection-risk data	Moderate, guideline-based	Postpone elective CIED implantation until fever and clinical signs of infection have resolved
Alcohol-based skin antisepsis	Randomized controlled trials (non-CIED and CIED), guideline documents	Moderate	Use alcohol-based preparation routinely
Adhesive incise drapes	Randomized controlled trials, meta-analyses, and observational studies	Low to moderate	Avoid non-antimicrobial drapes; consider iodine-impregnated drapes in selected high-risk procedures
Venous access strategy	Observational studies and meta-analyses	Low to moderate, indirect	Prefer cephalic or axillary access when feasible to reduce access-related complications, lead failure, and downstream reintervention.
Antibacterial envelope in high-risk patients	Randomized controlled trials and guideline documents	Moderate to high	Consider in selected high-risk patients
Generator pocket optimization	Observational studies and expert consensus	Low	Avoid excessive tension or dead space; consider submuscular placement in low-BMI or fragile soft-tissue patients
Pocket irrigation strategies	Guideline documents and observational studies	Low	Perform vigorous saline irrigation; avoid routine local antibiotic or antiseptic instillation
Suture material and wound closure optimization	Surgical RCTs, meta-analyses, observational/mechanistic data	Low to moderate, indirect	Ensure atraumatic, tension-free layered closure; antimicrobial-coated sutures may be considered, recognizing limited CIED-specific evidence

The strength of evidence was assigned narratively by the authors according to the hierarchy, consistency, and directness of the available evidence. It should not be interpreted as equivalent to formal guideline classes of recommendation or levels of evidence. CIED, cardiac implantable electronic device; DOAC, direct oral anticoagulant; HbA1c, glycated haemoglobin; RCT, randomized controlled trial; VKA, vitamin K antagonist.

**Table 2:** Major risk factors for CIED infection, categorized according to their theoretical modifiability at the time of device implantation.

Patient-Related	Device-Related	Procedure-Related
*Modifiable*		
Active systemic or local infection at the time of implantation, *OR 4.27* [16] Active skin disorders at the implantation site, *OR 2.46* [16]		Absence of perioperative antibiotic prophylaxis [16,17,18,19] Prolonged procedure duration [16,20,21,22] Postoperative pocket haematoma, *OR 8.5* [16] Inexperienced operator, *OR 2.9* [16] Temporary pacing, *OR 2.3* [16] Heparin bridging, *OR 1.9* [16] Oral anticoagulants, *OR 1.6* [16]
*Partly modifiable*		
Diabetes mellitus, *OR 2.08* [16]	Epicardial leads, *OR 8.09* [16] Abdominal generator pocket, *OR 4.01* [16] ≥2 leads, *OR 2.02* [16] Dual-chamber device, *OR 1.45* [16] Abandoned leads or complex lead route [1,6]	Reintervention for lead dislodgement, *OR 6.37* [16] Device replacement or revision, *OR 1.98* [16,22,23]
*Non-modifiable*		
End-stage renal disease, *OR 8.73* [16] History of previous device infection, *OR 7.84* [16] Corticosteroid therapy, *OR 3.44* [16] Renal insufficiency, *OR 3.02* [16] Chronic obstructive pulmonary disease, *OR 2.95* [16] NYHA functional class ≥ II, *OR 2.47* [16] Malignancy, *OR 2.23* [16] Young age [16,21] Male sex [16,21] BMI > 30 [16]		

BMI, body mass index; CIED, cardiac implantable electronic device; NYHA, New York Heart Association functional class; OR, odds ratio.

**Table 3:** Periprocedural management of antithrombotic therapy in CIED implantation.

Antithrombotic Therapy	Recommended Periprocedural Strategy
Aspirin monotherapy	Continue throughout the procedure
Dual antiplatelet therapy (DAPT)	>1 month after PCI and >6 months after ACS continue aspirin, interrupt P2Y12 inhibitor (ticagrelor ≥ 3 days, clopidogrel ≥ 5 days, prasugrel ≥ 7 days)
	<1 month after PCI or <6 months after ACS postpone elective CIED implantation; if urgent, continue DAPT
Vitamin K antagonists (VKA)	Continue VKA with INR in therapeutic range (2–3.5)
Direct oral anticoagulants (DOACs)	Continue or temporarily interrupt per operators preference If interrupted: Perform procedure at trough DOAC levels (12 h for twice-daily DOACs; 24 h for once-daily DOACs) Consider longer interruption (24–48 h) in patients with impaired renal function, higher bleeding risk, or undergoing device revision or upgrade
OAC + antiplatelet therapy	Continue OAC (VKAa or NOAC) Discontinue antiplatelet per patient-specific risk/benefit analysis
Heparin bridging (UFH or LMWH)	Avoid

Based on evidence from the BRUISE CONTROL trials, the 2021 EHRA Practical Guide on the use of non-vitamin K antagonist oral anticoagulants, and the 2021 ESC Guidelines on cardiac pacing and cardiac resynchronization therapy [37,38,39,40]. ACS, acute coronary syndrome; CIED, cardiac implantable electronic device; DAPT, dual antiplatelet therapy; DOAC, direct oral anticoagulant; INR, international normalized ratio; LMWH, low-molecular-weight heparin; OAC, oral anticoagulant; PCI, percutaneous coronary intervention; UFH, unfractionated heparin; VKA, vitamin K antagonist.

**Table 4:** Recommended agents, dosing, timing, and redosing intervals for antibiotic prophylaxis in cardiac implantable electronic device implantation [18].

Agent	Standard Dose	Timing	Redosing Interval (from Initiation of Preoperative Dose)
Cefazolin	2 g (3 g ≥ 120 kg) i/v	≤60 min before incision	>240 min
Cefuroxime	1.5 g i/v	≤60 min before incision	>240 min
*Alternative Agents in patients with β-Lactam Allergy*			
Clindamycin	900 mg i/v	≤60 min before incision	>360 min
Vancomycin	15 mg/kg	≤120 min before incision	N/A
*Documented methicillin-resistant Staphylococcus aureus (MRSA) colonization*			
Vancomycin	15 mg/kg	≤120 min before incision	N/A

**Table 5:** Most commonly used antiseptic agents for preoperative skin preparation [33].

Antiseptic Agent	Formulation	Key Considerations
Chlorhexidine–alcohol	2% CHG in 70% isopropyl alcohol	Rapid action, residual activity strongest evidence for SSI reduction among commonly used agents Rare risk of skin irritation Highly flammable, must be allowed to dry by evaporation
Povidone–iodine (alcohol-based)	10% PVP-I in 70% isopropyl alcohol	Broad antimicrobal spectrum Rapid onset due to alcohol, limited residual activity Iodine allergy should be considered Highly flammable, must be allowed to dry by evaporation
Povidone–iodine (aqueous)	10% PVP-I in water	Slower onset, no residual activity Iodine allergy should be considered

CHG—chlorhexidine gluconate; PVP-I—povidone–iodine; SSI—surgical site infection.

**Table 6:** Characteristics of Suture Materials [68,69].

Suture Type	Technical Parameter	Potential Advantages	Potential Limitations
*by structure*			
Monofilament	Single filament	Smooth surface enabling atraumatic tissue passage; lower bacterial adherence; reduced tissue reactivity	Reduced knot security; increased tendency to slip; comparatively less favorable handling
Multifilament	Multiple filaments braided or twisted	Higher tensile strength; improved handling and knot security	Increased surface area may facilitate bacterial colonization; higher tissue friction during passage
*by biological feature*			
Absorbable	Gradually degraded (hydrolysis or enzymatic)	Smooth wound approximation; gradual absorption limiting prolonged foreign body reaction; no need for suture removal	Temporary tensile strength; variable degradation time
Non-absorbable	Persistent in tissue	Durable fixation; commonly used for lead and generator anchoring	Permanent foreign material; potential for chronic tissue response

## Data Availability

No new data were created or analyzed in this study.
